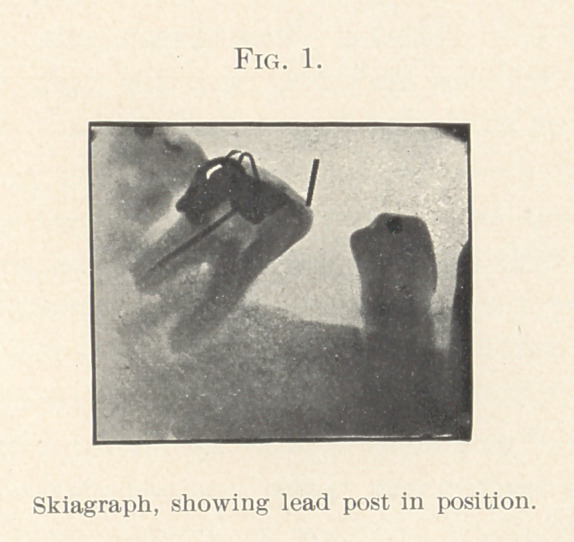# What Is the Meaning of Conservatism in Dental Practice?

**Published:** 1903-07

**Authors:** C. Edmund Kells

**Affiliations:** New Orleans, La.


### WHAT IS THE MEANING OF CONSERVATISM IN DENTAL PRACTICE?

BY C. EDMUND KELLS, JR., D.D.S., NEW ORLEANS, LA.

THE March issue of the INTERNATIONAL DENTAL JOURNAL contains a paper by Dr. J. G. Palmer, of New York, which was read before the Academy of Stomatology in October last, and the title of which was "Conservatism *versus* Radicalism in Certain Dental Operations."

A perusal of this article leads one to ask, What is the meaning of the term "conservatism" in dental practice? Does it mean to everlastingly adhere to methods once adopted and never to change? And what should be the proper interpretation of the word "radicalism"? Is it changing from *original methods?*

These are evidently the essayist's interpretations of these words, but I challenge their accuracy. If such were universally accepted, which fortunately is not so, the end of all improvement would be at hand.

I hold that "conservatism" means the adoption of apparently questionable new methods which differ radically from the old, in favorable cases which are expected to be under the control of the operator and the results of which may be observed. Once this period of probation is satisfactorily passed, the radical operation is such no longer, but becomes an accepted method and should always be practised in a conservative manner.

"Radicalism," to me, means the adoption of an entirely new operation or method, entirely upon the "say so" of some one else, and performing said operation at every opportunity, practically without regard to consequences.

A most beautiful illustration of this differentiation was demonstrated by the introduction of the operation of implantation by Dr. Younger. For a year or two after his discovery, at numerous dental conventions, many operators of the *bona fide* "radical kind" gave clinics of implantation, performing the operation without any idea of conservatism, and most of them probably making a clean score of one hundred per cent. of failures.

The conservative operators selected their cases, which were few and far between, observed the causes of the failures of others, as well as their own, and met with success sufficient to warrant the operation being a conservative one in their hands to-day, and one which nothing else has replaced.

The following is a quotation in the paper in question:

"We have sometimes failed in accomplishing what another has claimed to be an easy matter, because that one has omitted from his description some detail which he took for granted we were conversant with." And now I will quote from the essayist: "There are those who earnestly advocate what they term immediate root-filling. This, as I understand it, means that a devitalized tooth is opened, cleaned, sterilized, and filled at one sitting."

Here, in these two sentences, is the key-note to the whole situation, and accounts for the essayist's acknowledgment that his experience in immediate root-filling has been "unfortunate," as he expresses it. In the first sentence the failure in an operation is attributed to the assumption of knowledge of some unmentioned detail; and in the second, the sequence of the necessary steps for the successful practice of immediate root-filling is given as opening, cleaning, sterilizing, and filling. Mind you, how particular each step is emphasized, *open, clean, sterilize,* and *fill.* Well, those of you who have never practised the "radical" operation of immediate root-filling, follow those instructions to the letter, and likewise will your experience be "unfortunate," for the most important "detail" of the operation is omitted.

Of course, I speak from the stand-point of my own practice, and having practised immediate root-filling for nearly twenty years, during which time it has never occurred to me to "treat" a tooth found filled with pus and no fistula, any more than I would "treat" a small coronal cavity to be filled with gold.

The steps in the two operations are precisely alike. Note them: Open, that is prepare the cavity; cleanse, that is remove the decay, débris, and foreign matter; sterilize, the small cavity is sterilized just the same as the root-canal; dry, d-r-y,—that is the feature upon which the success of the operation depends. If I cannot dry the small coronal cavity, I do not fill with gold. If I cannot dry the root-canal, I do not fill it. That is the only requisite I demand which is sometimes beyond my control. I can always "open" the cavity, but I find occasionally a root-canal which I cannot dry. Under those circumstances it cannot be cleaned or sterilized, consequently it *cannot* be filled properly, and such is not attempted. Probably at the next sitting this may be successfully done.

I hold that, no matter what the condition of the tooth at the beginning of the operation, if the root-canal can be gotten perfectly dry, that is the best time to fill it, for treat it until doomsday and you will not improve its condition.

As I said before, I am merely giving you my views. The essayist quoted freely from Dr. F. Milton Smith. Notwithstanding not having had the pleasure of meeting Dr. Smith, I would risk my reputation upon his being a careful, skilful, conservative operator, and one to whom the most minute detail always appears of the greatest importance, and I venture to say his drying his canals is his "sheet-anchor" in this turbulent proposition; in fact, in the discussion of the paper he states as much.

In the discussion of the paper the essayist says that to his knowledge, during a practice of twenty-five years, he has lost but two teeth under his "continuous performance" method. Candor, for which I have a reputation, will not allow me to give my record, as it would subject me to the mortification of an unfavorable comparison with the above, but I am confident that I now have less trouble with the immediate method than I had previously with the other.

Let me cite a recent and fairly typical case:

Mr. O. P., aged about fifty years, called, complaining of trouble with a right lower second molar, which was painful under pressure and very loose. The tooth had been troubling him for some six months or more, during all of which time it had been under "treatment." It would be "treated" for a period, when it would be sealed temporarily, which sealing was always followed by pain, and conse-

Fig. 1.

Skiagraph, showing lead post in position.

quently removed and the "treatment" continued. Finally becoming tired of all this "treatment," he decided to come to me.

An appointment was given, the tooth well opened up, when the remains of two anterior canals were found very fine, and probably about half the length of the root. The posterior canal was large, with evidently something very wrong at the end of the root, as a large opening was found at a depth much less than the length of the root was judged to be, perforation, therefore, being surmised, from which a large quantity of pus came. As usual in all such cases, a lead post was carefully fitted just to reach this perforation and secured in position, when a skiagraph was taken with the result as shown. (Fig. 1.)

From this it was clearly seen that this root had been considerably absorbed, that the lead post just reached to its apex, and that considerable diseased tissue surrounded the apices of each root. Everything being now clear sailing, the root-canals were filled as usual, *while the tooth was still sore and loose,* and immediate improvement set in, as was expected. In a few days all soreness had disappeared, and when I saw the patient a few months later, it had become quite solid and had resumed its function of being a very useful tooth, and was to all appearances in perfect condition.

Here is a fair sample of how a tooth was being "treated" to death, but fortunately rescued, not by any radical procedure, but by good common-sense conservative methods, which were by no means new, and it does seem that such methods, having about obtained their majority in point of age, should supersede such practice as advocated by your essayist, and that the pages of our dental journals should no longer be wasted in accounts of what should be practically considered obsolete methods of the management of pulpless teeth.

[Remarks.—The author of this paper wishes an opinion of this case. There probably can be but one given,—that in cases unyielding, such as this, immediate filling should be attempted. The writer has frequently performed similar operations with varying results, but even where these are of the best they do not affect a general principle. The author evidently abandons his former statement, and the one which resulted in this paper, by insisting on a *dry canal.* There will, probably, be no dispute as to this being generally a very safe canal for immediate filling.—Ed.]

## Figures and Tables

**Fig. 1. f1:**